# Clinicopathological Factors Affecting Prognosis in Patients with Advanced Cervical Cancer Undergoing Concurrent Chemoradiation Therapy

**DOI:** 10.3390/cancers17183042

**Published:** 2025-09-18

**Authors:** Maitreyee Parulekar, Min Kyung Kim, Joseph J. Noh, Dong Hoon Suh, Kidong Kim, Yong Beom Kim, Jae Hong No

**Affiliations:** 1 Department of Obstetrics and Gynecology, Seoul National University Bundang Hospital, Seongnam 13620, Republic of Korea; drmaitreyeeparulekar@gmail.com (M.P.); juventa0412@snu.ac.kr (M.K.K.); josephnoh@snu.ac.kr (J.J.N.); sdhwcj@snubh.org (D.H.S.); kidong.kim.md@snubh.org (K.K.); ybkimlh@snubh.org (Y.B.K.); 2 Department of Minimally Invasive Gynecology and Gynecological Oncology, Galaxy Care Institute, Pune 411004, India; 3 Department of Obstetrics and Gynecology, Seoul National University College of Medicine, Seoul 03080, Republic of Korea

**Keywords:** cervical cancer, concurrent chemoradiation, prognostic factor, BMI, para-aortic node

## Abstract

Background: Concurrent chemoradiation therapy (CCRT) is the standard treatment for patients with locally advanced cervical cancer (LACC), including those with parametrial or lymphatic metastasis. However, therapeutic outcomes vary, and prognostic factors remain inadequately defined. Methods: We conducted a retrospective study involving 128 patients with cervical cancer who received definitive CCRT between 2003 and 2022 at Seoul National University Bundang Hospital. We evaluated clinicopathological variables, including age, height, body weight, histologic type, tumor size, human papillomavirus (HPV) type, squamous cell carcinoma (SCC) antigen levels, and involvement of the parametrium, lower vagina, and lymph nodes. Survival outcomes were analyzed using Kaplan–Meier curves and Cox proportional hazards models. Results: Stage IIIC1r, according to the 2018 FIGO staging system, was the most common disease stage among the study population. Para-aortic lymph node metastasis was significantly associated with increased recurrence risk (odds ratio [OR] = 5.892; 95% confidence interval [CI]: 2.030–17.097; *p* = 0.001) and was linked to poorer progression-free survival (PFS, *p* = 0.001), overall survival (OS, *p* = 0.014), and treatment-free interval (TFI, *p* = 0.001). Obesity (body mass index ≥ 25 kg/m^2^) was also associated with higher recurrence risk (OR = 2.737; 95% CI: 1.093–6.855; *p* = 0.032) and reduced PFS (*p* = 0.0089). Conclusions: Para-aortic lymph node metastasis and obesity are significant prognostic factors in patients undergoing definitive CCRT for LACC. These findings highlight the need for risk-adapted treatment strategies and potential incorporation of adjunctive therapies to improve clinical outcomes in high-risk patient subgroups.

## Simple Summary

This study examined factors that influence treatment outcomes in women with advanced cervical cancer who were treated with concurrent chemoradiation therapy (CCRT), the current standard of care. By analyzing data from 128 patients treated between 2003 and 2022 at a single institution, researchers found that the presence of cancer in the para-aortic lymph nodes was strongly linked to a higher risk of cancer recurrence and lower survival rates (5-year survival was only 60% as compared to 89% in women without spread). Additionally, obesity (body mass index ≥ 25 kg/m^2^) was associated with worse treatment outcomes, including a higher risk of recurrence and shorter treatment-free intervals. Pre-treatment SCC antigen showed a minor effect on survival. These findings highlight the importance of identifying high-risk patients who may benefit from more personalized or intensified treatment strategies alongside standard CCRT.

## 1. Introduction

Globally, cervical cancer ranks fourth in terms of female incidence and mortality (7.7% of all cancer-related deaths in women), with ~660,000 new cases reported in 2022 [1,2]. These figures are unacceptable, given that most cervical cancer cases are attributed to the human papilloma virus—a preventable disease with well-established screening and vaccination programs [3]. In the Republic of Korea, the incidence of cervical cancer is steadily decreasing but remains the third leading cause of death in women [4]. Hence, there is an urgent need to develop effective treatment protocols. Early-stage disease can be cured by surgery and tailored adjuvant treatment; however, for locally advanced cervical cancer (LACC), according to the revised International Federation of Gynecology and Obstetrics (FIGO) staging system, the standard of care is definitive platinum-based concurrent chemoradiotherapy (CCRT) [5,6]. Multiple large-scale randomized trials have demonstrated that CCRT was associated with reduced recurrence and risk of death in LACC compared to RT alone [7,8,9]. Neoadjuvant chemotherapy followed by surgery represents an alternative approach; however, CCRT results in better disease-free survival in patients with LACC [9,10].

Approximately one-third of patients treated with CCRT experience relapse or disease progression during follow-up and show poor overall prognosis [11]. To identify better treatment strategies, various recent studies investigated the prognostic factors affecting cervical cancer based on disease stage and metastasis [12,13,14]. Hence, we conducted this retrospective study to explore the relevance of these prognostic factors in patients who received platinum-containing CCRT as a definitive treatment. We also investigated the associations between the independent prognostic factors and efficacy of CCRT, and determined whether high-risk patients could be identified for escalated therapy to improve their survival outcomes.

## 2. Materials and Methods

### 2.1. Case Selection

We retrospectively reviewed the data for 141 consecutive patients with cervical cancer who underwent definitive CCRT without any primary surgery between 2003 and 2022 at the Seoul National University Bundang Hospital (Seongnam, Republic of Korea). The updated 2018 FIGO staging system was implemented for cervical cancer staging, the patients before 2018 were also restaged as per the newer classification for the purpose of this study. We included patients (1) with invasive cervical cancer diagnosed by histopathology of punch biopsy specimens, (2) who received definitive CCRT and completed the treatment, and (3) with no previous surgery or any adjuvant treatment for cervical cancer. Of the 141 patients, 13 were not eligible and were excluded from the study. Therefore, 128 patients with stage IB2–IVA diseases were included in the statistical analysis. This study was approved by the Institutional Review Board of the Seoul National University Bundang Hospital (IRB No: B-2406-909-102). Informed consent was not required, given the retrospective nature of the study.

### 2.2. Clinicopathological Data Collection

We collected data on age, height, weight, BMI, histological type, maximal tumor diameter, human papillomavirus (HPV) infection, serum squamous cell carcinoma (SCC Ag) antigen levels, parametrium and lower vagina involvement, and lymph node (LN) metastasis (based on imaging analysis) from the hospital database. Pathological parameters were collected from data after surgery, all other factors have been collected from pre-operative data. LN metastasis was confirmed by a short-axis diameter ≥ 15 mm in T2w magnetic resonance imaging or computed tomography, or increased fluorodeoxyglucose uptake in positron emission tomography scans. Recurrence and survival data were also obtained.

### 2.3. Definitive Concurrent Chemoradiotherapy Procedure

The institutional standard for concurrent chemoradiation therapy (CCRT) involved external beam radiotherapy (EBRT) delivered concomitantly with weekly cisplatin chemotherapy, followed by definitive brachytherapy. EBRT was administered to the planning target volume (PTV) at a total dose of 45–50.4 Gy in 25–28 fractions (1.8–2.0 Gy per fraction, five fractions per week). In cases with radiological or histopathological suspicion of para-aortic lymph node involvement, extended-field EBRT (EF-EBRT) was employed to encompass the para-aortic nodal regions. Concurrent chemotherapy consisted of cisplatin at a dose of 40 mg/m^2^ administered intravenously once weekly for a total of 4 to 6 cycles, subject to patient tolerance and renal function.

Brachytherapy was initiated either during the final weeks of EBRT or immediately thereafter, utilizing either a conventional two-dimensional Point A–based technique or a three-dimensional image-guided approach based on CT or MRI. High-dose-rate (HDR) brachytherapy was delivered in 4–5 fractions of 6–7 Gy each, with the goal of achieving a total equivalent dose in 2 Gy fractions (EQD2) of 80–90 Gy to the high-risk clinical target volume (HR-CTV). Additional radiation boosts to involved lymph nodes or parametrial disease, as well as modifications to the chemotherapy or radiotherapy protocol, were made at the discretion of the treating oncologist and in accordance with recommendations from a multidisciplinary tumor board.

### 2.4. Follow-Up

The post-CCRT follow-up data of all patients, including clinical findings, serum tumor markers, and imaging results, were retrospectively reviewed until their last hospital visit/death, up to December 2023. Standard follow-up protocol of the institute included tumor marker testing every 3 months and CT scan every 5–6 months for the first 2 years and then double the interval for the next 3 years, annually thereafter. Individualization for follow-up protocol was performed for patients if needed, as per consultant’s decision. Response to treatment was classified as complete remission (CR), partial remission (PR), stationary disease, or progressive disease (PD) according to the Response Evaluation Criteria in Solid Tumors (RECIST) 1.1 [15]. Progression-free survival (PFS) was defined as the duration between the date of initial diagnosis and date of proven recurrence/tumor progression. Overall survival (OS) was defined as the time between the initial diagnosis by biopsy and death/last follow-up. The treatment-free interval (TFI) was defined as the duration between the dates of the last treatment and recurrence or second-line treatment.

### 2.5. Statistical Analysis

Clinicopathological variables were categorized into binomial groups, and basic statistical tests were used to describe tumor characteristics. Continuous data are described as median values and ranges whereas categorical data are presented as numbers and percentages. Logistic regression analyses (univariate and multivariate) were used to determine the association between independent variables and recurrence, along with odds ratios (ORs) and 95% confidence intervals (CIs). Survival analysis was performed using the Kaplan–Meier method, plots were generated including 95% CI shading and number-at-risk tables. The primary endpoints of the analyses were PFS, OS, and TFI. Cox proportional hazard regression models were used to evaluate the influence of independent factors on survival, along with hazard ratios (HRs) and 95% CIs. Proportional hazards assumptions were verified using Schoenfeld residuals; minor violations observed for age were addressed by stratification for PFS and TFI in the final Cox regression models. Body mass index (BMI), tumor size and SCC antigen levels were analyzed both as categorical and continuous variables to minimize information loss. As part of sensitivity analyses, an exploratory subgroup comparison was performed for underweight patients versus those with normal BMI. Forest plots were generated to visualize HRs from the multivariate Cox models. Statistical analyses were performed using SPSS version 25.0 (IBM SPSS, Armonk, NY, USA) and R version 4.3.0 for advanced survival graphics. Statistical significance was set at *p* < 0.05.

## 3. Results

A total of 128 patients were enrolled in the study. Clinicopathological characteristics are described in Table 1. Patients were divided into two groups according to age: <50 years (27 patients, 21.1%) and ≥50 years (101 patients, 78.9%). The body mass index (BMI) of the patients was divided into two groups: <25 kg/m^2^ (87 patients, 68%) and ≥25 kg/m^2^ (41 patients, 32%) as per the normal BMI cut-off [16]. As per FIGO staging, type IIIC1r was the most common disease, accounting for 60 cases (46.9%), 33 cases were IIB (25.8%), and 22 cases were IIIC2r (17.2%). Tumor size was ≥4 cm in 100 cases (78.1%) and <4 cm in 28 cases (21.9%). Amongst the pathological subtypes, SCC Ag was observed in 114 cases (89.1%) and non-SCC in 14 cases (10.9%). In total, 123 (96.1%) patients had underlying HPV infections. Imaging analysis revealed 88 (68.8%) patients with metastatic LNs (pelvic and/or para-aortic), whereas 40 (31.3%) had no LN metastasis. Para-aortic LN (PALN) metastasis was observed in 24 patients (18.8%), all of whom received radiation therapy in the para-aortic area (extended field radiotherapy). Treatment adherence as per the radiotherapy and chemotherapy modality was also studied as documented in Table S1. Adverse events experienced by the patients were recorded, with most common being anemia (83.6%) followed by nausea (59.4%) and thrombocytopenia (38.3%). Grade 3 and higher events were rare, with anemia and thrombocytopenia each at 3.9%. (Table S1). Amongst the study population, 44.5% (*n* = 57) had anemia, 33.6% of patients had hypertension (*n* = 43) and 7% (*n* = 9) had diabetes mellitus (Table S1).

Disease recurrence occurred in 35 (27.3%) patients, which was further classified as local, regional, or distant, depending on the site of recurrence [17]. Of these, 6/35 patients had locoregional disease with no distant spread. Most (29/35 patients, 82.85%) showed recurrence at distant sites, including PALNs and distant organs, such as the lung and liver, with or without intrapelvic disease (Table 2). Complete remission of the disease after CCRT was observed in 107 patients (83.6%), whereas partial remission was noted in 16 patients (12.5%). The disease progressed despite CCRT in one patient (0.8%).

The univariate logistic regression analysis showed that BMI ≥ 25 kg/m^2^ (OR: 2.737; 95% CI: 1.093–6.855; *p* = 0.032) and para-aortic metastasis (OR: 5.892; 95% CI: 2.030–17.097; *p* = 0.001) were significantly associated with greater odds of recurrence (Table 3a). There were no associations between recurrence and patient age (OR: 0.994; 95% CI: 0.345–2.868: *p* = 0.991), tumor size (OR: 2.394; 95% CI: 0.720–7.694; *p* = 0.154), or pelvic LN metastasis (OR: 1.584; 95% CI: 0.537–4.673; *p* = 0.405).

Multivariate logistic regression analysis was performed to investigate factors independently associated with higher recurrence rates (Table 3b). The results were similar to those of the univariate analysis in that greater BMI (OR: 2.697; 95% CI: 1.087–6.692; *p* = 0.032) and PALN metastasis (OR: 6.931; 95% CI: 2.540–18.916; *p* < 0.001) were associated with recurrence, whereas tumor size ≥ 4 cm showed no association.

**Figure 1:**
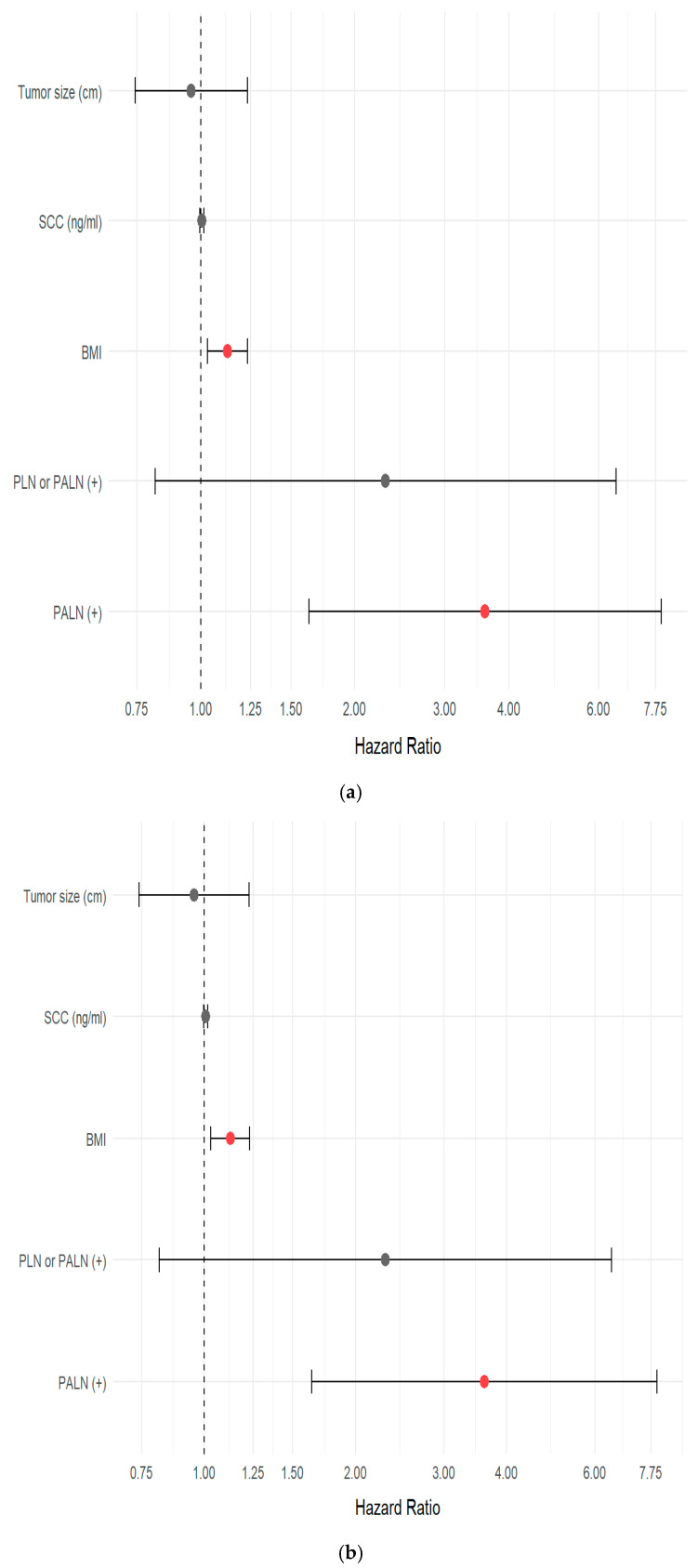

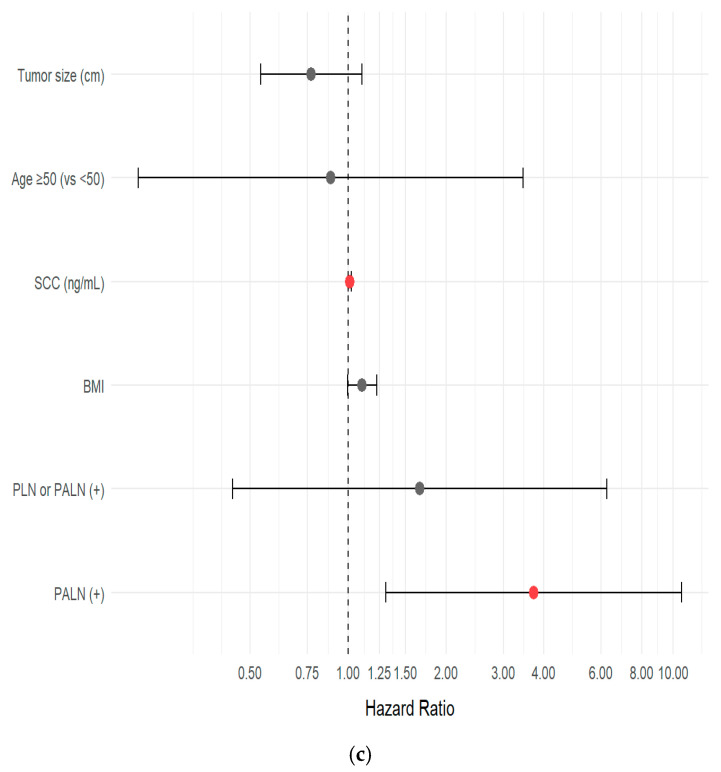
(**a**) Forest Plot for PFS, (**b**) Forest plot for TFI, (**c**) forest plot for OS.

**Figure 2:**
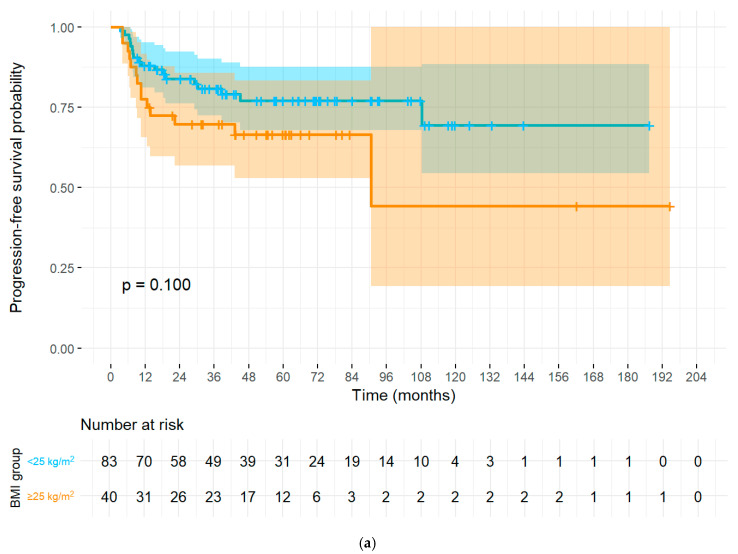

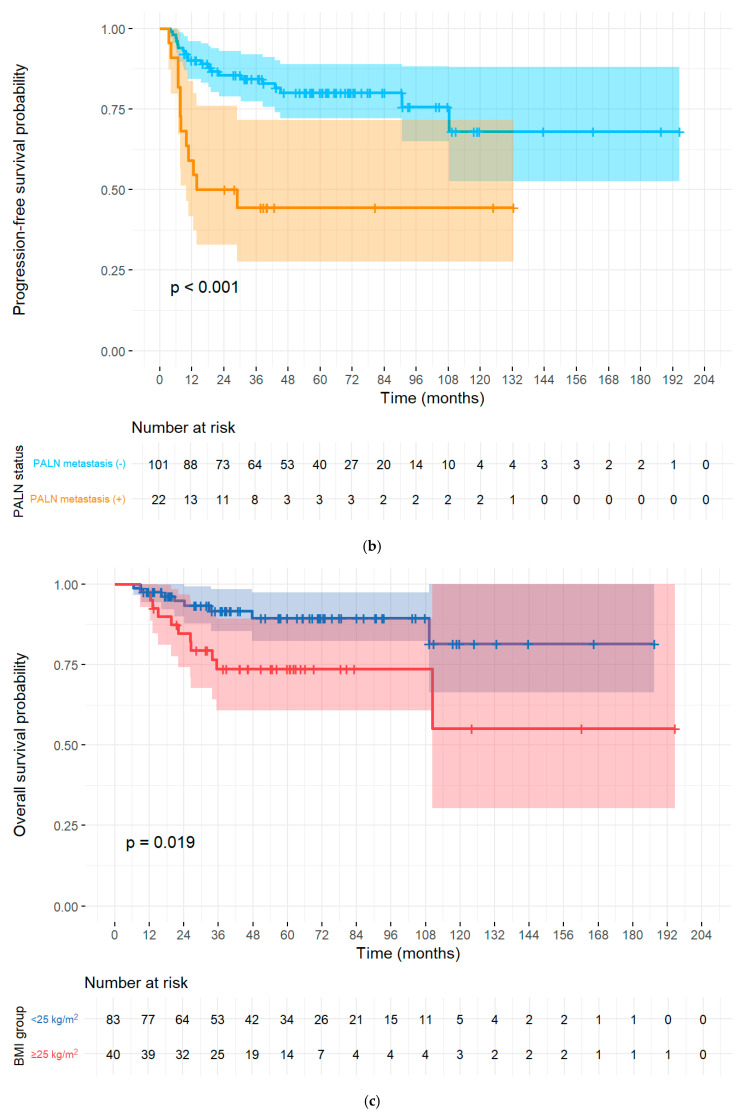

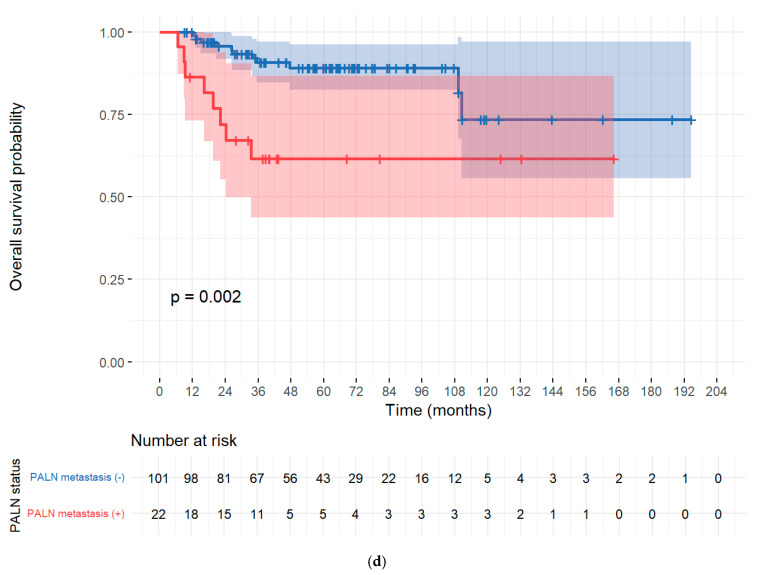
(**a**) Kaplan–Meier Curve (PFS by BMI), (**b**) Kaplan–Meier Curve (PFS by PALN metastasis), (**c**) Kaplan–Meier Curve (OS by BMI), (**d**) Kaplan–Meier Curve (OS by PALN).

The PALN metastasis remained the strongest adverse factor across endpoints, with multivariable, age-stratified Cox models showing PALN positivity associated with markedly worse PFS (HR 3.59, 95% CI 1.63–7.93, *p* = 0.0015) and TFI (HR 3.61, 95% CI 1.64–7.97, *p* = 0.0015). BMI (continuous) independently worsened PFS and TFI (PFS: HR 1.13 per kg/m^2^, 95% CI 1.03–1.23, *p* = 0.0089; TFI: HR 1.13 per kg/m^2^, 95% CI 1.03–1.23, *p* = 0.0092). For OS (no age stratification required by PH testing), PALN positivity was independently prognostic (HR 3.72, 95% CI 1.30–10.60, *p* = 0.0141), BMI (continuous) showed a borderline adverse association (HR 1.10 per kg/m^2^, 95% CI 0.99–1.22, *p* = 0.0628), and SCC antigen had a small but significant effect (HR 1.0107 per unit, 95% CI 1.0005–1.0211, *p* = 0.0398). The multivariate cox regression analyses for OS, PFS, and TFI using the Cox proportional hazards model are summarized in Table 4 with graphical representation in forest plots for the same (Figure 1a–c). Kaplan–Meier curves used for PALN and BMI categories with number-at-risk tables and 95% CI shading, visually mirroring the Cox findings (Figure 2a–d). Total dose of radiotherapy or co-morbidities like diabetes, hypertension or initial pre-treatment hemoglobin levels did not show any significant associations with PFS or OS (Table S1).

## 4. Discussion

Predictive models of cancer outcomes have gained widespread interest in recent years for their predictive power of patient responses to certain treatments [18]. These models would allow for the development of targeted interventions tailored to independent prognostic factors, such as patient demographics, tumor size, histological type, LN metastases, low hemoglobin levels, and FIGO staging [12,13,14]. In our study, BMI and PALN metastasis were key independent prognostic factors in patients with cervical cancer who underwent primary CCRT.

Most patients in the current study presented with stage IIIC1r disease (46.9%), followed by stage IIB (25.8%). Of the 128 patients, 122 (95.3%) had parametrial invasion, whereas only two (1.56%) had lower vaginal involvement and none presented with stage IIIA disease. FIGO 2018 has better survival discriminatory capacity than FIGO 2009, and stage III is more commonly encountered at disease presentation [14,19]. Grigsby et al. [19] showed that survival in stage III subclassifications was heterogeneous—IIIC1 showed better survival than IIIB; however, other retrospective studies showed comparable outcomes for IIIB and IIIC1, but significantly poorer outcomes for IIIC1 [9,14]. This emphasizes the importance of factors other than FIGO staging in disease prognosis, such as the extent of tumor invasion into the surrounding structures other than LN metastasis, which may better inform the risk analysis for personalized treatments. We could not assess the association between lower-third vaginal involvement and recurrence or survival because of the small sample size. Nevertheless, parametrial invasion in the univariate analysis did not show any significant association with recurrence.

Para-aortic lymph node metastasis was consistently associated with inferior survival outcomes, with significantly worse PFS (HR 3.60, 95% CI 1.62–8.01, *p* = 0.0015), OS (HR 3.73, 95% CI 1.31–10.61, *p* = 0.014), and TFI (HR 3.58, 95% CI 1.61–7.98, *p* = 0.0016) compared with PALN-negative patients (Table 4). Pelvic LN metastasis, however, did not emerge as a significant prognostic factor in our analysis. This finding may partly reflect the unequal distribution between comparison groups, which could have limited the statistical power to fully assess the prognostic implications of pelvic nodal disease. Several studies have confirmed that larger and more numerous LNs are associated with worse prognosis in cervical cancer [20,21,22]. In-field recurrence was high in patients with large LN metastases, as previously reported [23]. In our study, the majority of patients with recurrent disease had distant metastasis with or without intrapelvic disease. Among the distant sites of recurrence, PALNs were the most common. This suggests a need for escalated adjuvant or neoadjuvant treatment, in addition to definitive CCRT, to improve survival outcomes in patients with high-risk LN characteristics. In these patients, LN-directed volumetric modulated arc therapy or simultaneous integrated boost with intensity-modulated radiation therapy may inhibit locoregional recurrence [24]. Additional chemotherapy may also improve the survival outcomes in such cases. The GCIG INTERLACE randomized trial showed that short-course weekly induction chemotherapy before standard CRT improved PFS and OS in LACC [25]. Pre-treatment surgical resection of bulky LNs could limit the adverse effects related to increased radiation or anticancer drug dosage and may improve survival outcomes in selected patients [23]. In this regard, the results of the ongoing DEBULK trial (ClinicalTrials.gov Identifier: NCT05421650) are awaited. Immune checkpoint inhibitors are a promising therapeutic strategy against LACC [26]. The recent randomized controlled trial KEYNOTE A18 showed that pembrolizumab plus chemoradiotherapy significantly improved PFS in patients with high-risk LACC [27]. The ongoing EMBRACE II study represents the next step in personalized radiotherapy for locally advanced cervical cancer, incorporating MRI-guided brachytherapy and advanced IMRT/IGRT with risk-adapted dose prescriptions to improve tumor control while minimizing toxicity [28]. Importantly, EMBRACE II also integrates imaging and biomarker research to refine prognostication. These efforts parallel our findings, underscoring the need to combine patient-level factors such as BMI with advanced radiotherapy strategies to further optimize outcomes in CCRT-treated patients.

A recent study on the effects of sarcopenic obesity on cervical cancer indicated a strong association between BMI, PFS, and OS, with normal-weight patients showing the highest survival rates [18]. Gnade et al. [29] and Clark et al. [30] also reported worse OS in both underweight and obese individuals with LACC. In our analysis, BMI ≥ 25 showed strong prognostic value. On continuous analysis, each 1 kg/m^2^ increase was associated with a 13% higher risk of progression (HR 1.13, 95% CI 1.03–1.24, *p* = 0.009) and a 12% higher risk of earlier recurrence (TFI: HR 1.12, 95% CI 1.02–1.23, *p* = 0.017). A borderline adverse effect was observed for OS (HR 1.10, 95% CI 1.00–1.22, *p* = 0.06). When analyzed categorically, patients with BMI ≥ 25 had significantly worse OS than those with BMI < 25 (log-rank *p* = 0.03). An exploratory subgroup analysis of underweight patients (BMI < 18.5, *n* = 5) did not show significantly worse PFS (log-rank *p* = 0.60) or OS (log-rank *p* = 0.70) compared with normal BMI (Table S2). However, no recurrences or deaths occurred in this very small group, limiting interpretation. The influence of body composition, particularly body mass index (BMI), on cervical cancer outcomes is increasingly recognized, though the biological underpinnings remain incompletely defined. Obesity contributes to tumor progression not only through excess adiposity but also via a chronic inflammatory state characterized by elevated cytokines (e.g., IL-6, TNF-α) and adipokines such as leptin. These mediators can activate signaling cascades including JAK/STAT and PI3K/Akt/mTOR, drive angiogenesis, and create an immune-suppressive milieu that reduces the effectiveness of chemoradiation. Pharmacologically, obesity alters the distribution and metabolism of cytotoxic agents, potentially increasing toxicity or necessitating dose adjustments, while in radiation therapy, body habitus may affect pelvic dose delivery [18]. Tumor hypoxia decreases radiosensitivity by limiting oxygen-dependent DNA damage and activating survival pathways, and obesity exacerbates this radiation resistance by fostering a hypoxic, inflamed, and immunosuppressed tumor microenvironment. In addition to obesity alone, sarcopenic obesity—the coexistence of adiposity and low skeletal muscle mass—appears particularly detrimental. The strength of our study is that we extended our analysis to the effect of BMI in CCRT-treated patients other than the standard prognostic factors, and we found robust evidence regarding its impact on prognosis. Going forward, more refined assessments of body composition, including visceral fat and muscle indices, are warranted. With the advent of imaging-based and AI-driven analyses, prospective studies can clarify whether targeted interventions to optimize body composition could enhance treatment response and long-term survival in locally advanced cervical cancer [18,29,31].

We also studied the associations between age, tumor size, or SCC antigen levels and recurrence or survival outcomes, but found no independent predictors in our cohort. However, there has been much controversy regarding these correlations [12,13]. Volgger et al. [32] found that SCC antigen levels < 2 ng/mL were associated with better OS (*p* < 0.001), whereas Chen et al. [12] (cut-off value 2) did not find any relationship. Our analysis showed that 84 patients (68.3%) had elevated serum SCC antigen levels (cut-off value 2), only a weak association with OS was observed when modeled continuously (HR 1.01, 95% CI 1.00–1.02, *p* = 0.04). Dattoli et al. [33] showed that patients aged < 40 years had poorer prognoses. This could be because, in younger patients, the proportions of neuroendocrine disease and adenocarcinoma was higher, with patients being more prone to LN and distant metastasis [2]. Similar studies did not find any differences in OS or PFS between the two age groups [9,34]. Our data had a similar age ratio (with 78.9% patients aged ≥ 50 years), and our results were comparable with most studies indicating no association with recurrence or OS, though there was a significant difference in the number of patients in the two groups. We observed strong differences in the number of patients with small and large tumors: only 28 (21.9%) patients had a tumor size < 4 cm, whereas 100 (78.1%) presented with a tumor size of ≥4 cm. This could be because the majority of patients in our cohort presented with stage III disease. In our analysis, there was no significant association between tumor size and recurrence/survival, as observed by Phung (cut-off 4 cm) [14]. Endo et al. [13] also found that tumor size had no prognostic relevance at > 5 cm, but tumors of 6 and 7 cm had an HR of 2.32 (95% CI: 1.21–4.43) and 2.53 (95% CI: 1.19–5.38), respectively, in a univariate analysis. To address the potential impact of advances in radiation techniques, supportive care, and imaging over the 20-year study period, we stratified patients by diagnosis year (before vs. after 2017) and performed Kaplan–Meier analyses for both PFS and OS. No significant differences were observed between the two time periods (PFS: log-rank *p* = 0.822; OS: log-rank *p* = 0.153), suggesting that these temporal changes did not materially influence survival outcomes in our cohort (Table S2). These discrepancies underscore the heterogeneity of locally advanced cervical cancer (LACC) cohorts and the need for multicenter validation.

We acknowledge the limitations of this study, including its single-center retrospective design, convenience sampling, and relatively small sample size. Another limitation is the lack of information on host immune status, such as baseline lymphocyte count, neutrophil-to-lymphocyte ratio, or other immune-related markers, which are increasingly recognized as important prognostic factors in cervical cancer. Future prospective studies should integrate immune-related parameters alongside clinical and body composition variables to provide a more comprehensive assessment of outcomes following CCRT. In addition, further evidence is required to establish the most effective therapeutic strategies for patients with bulky lymph nodes in LACC.

## 5. Conclusions

Our study represents a valuable contribution to ongoing efforts aimed at understanding the treatment outcomes and prognostic factors in patients with cervical cancer treated with CCRT. This information can inform the development of personalized CCRT-based treatments for selected patients with poor prognostic factors to improve OS outcomes. Our findings highlight an important research question: Should patients with higher BMI and radiologically enlarged PALNs receive adjunctive treatment in addition to CCRT? Further prospective research is needed to validate these prognostic factors, develop effective therapeutic strategies, and adaptively adjust treatment protocols in patients with LACC.

## Figures and Tables

**Table 1:** Clinicopathological features in patients with cervical cancer.

Variables	No. of Patients (*n* = 128)	Value (%)
Age		
Median	59 (32–87)	
<50	27	21.1
≥50	101	78.9
Body mass index (kg/m^2^)		
Mean	24.0 ± 3.9	
<25	87	68.0
≥25	41	32.0
FIGO stage (2018)		
IB2	1	0.8
IIA2	1	0.8
IIB	33	25.8
IIIB	6	4.7
IIIC1r	60	46.9
IIIC2r	22	17.2
IVA	5	3.9
Histology		
Non-SCC	14	10.9
SCC	114	89.1
Tumor size (cm)	Mean = 4.9 ± 1.4	
<4	28	21.9
≥4	100	78.1
HPV infection		
Negative	5	3.9
Positive	123	96.1
Lymph node metastasis		
Negative	40	31.3
PLN or PALN	88	68.8
PALN metastasis		
No	104	81.3
Yes	24	18.8
Parametrial invasion		
No	6	4.7
Yes	122	95.3
SCC Ag		
<2	39	31.7
≥2	84	68.3
Recurrence		
No	93	72.7
Yes	35	27.3

HPV—human papilloma virus, SCC Ag—serum squamous cell carcinoma antigen, PALN—para-aortic lymph node.

**Table 2:** Response and initial recurrence pattern in cervical cancer patients treated with CCRT.

Response to CCRT (*n* = 128)	Percent (%)
CR 107	83.6
PR 16	12.5
SD 4	3.1
PD 1	0.8
Recurrence Pattern (*n* = 35)	Number of patients
Local	2
Regional	4
Distant	29
Sites of Recurrence	Number of patients
Intrapelvic space (including pelvic lymph nodes)	14
Para-aortic Lymph nodes	11
Lung	9
Mediastinum	3
Liver	3
Bone	4
Other distant (SCN/Inguinal nodes/Axillary nodes/Psoas muscle)	14

CR—complete response, PR—partial response, SD—stable disease, PD—progressive disease, SCN—supraclavicular lymph nodes, CCRT—concurrent chemoradiotherapy.

**Table 3:** (**a**). Univariate logistic regression analysis of independent factors for recurrence. (**b**). Multivariate logistic regression analysis of independent factors for recurrence.

Variables		Recur		OR	95% CI	*p*-Value
		No	Yes			
Age	<50	20	7	0.994	0.345–2.868	0.991
	≥50	73	28			
BMI (kg/m^2^)	<25	67	20	2.737	1.093–6.855	0.032
	≥25	26	15			
Tumor size (cm)	<4	22	6	2.394	0.720–7.694	0.154
	≥4	71	29			
PLN or PALN metastasis	No	34	6	1.584	0.537–4.673	0.405
	Yes	59	29			
PALN metastasis	No	83	21	5.892	2.030–17.097	0.001
	Yes	10	14			
Parametrial invasion	No	4	2	0.742	0.130–4.241	0.737
	Yes	89	33			
SCC Ag	<2	28	11	0.848	0.361–1.994	0.706
	≥2	63	21			
**Variables**	**Beta**	**SE**		**OR**	**95% CI**	***p*-Value**
BMI ≥ 25 kg/m^2^)	0.992	0.464		2.697	1.087–6.692	0.032
Tumor size (≥4 cm)	0.946	0.594		2.574	0.804–8.239	0.111
PALN metastasis	1.939	0.512		6.931	2.540–18.916	<0.001

BMI—body mass index, PLN—pelvic lymph nodes, PALN—para-aortic lymph nodes, SCC Ag—serum squamous cell carcinoma antigen, OR—Odds ratio, CI—confidence interval. BMI—body mass index, PALN—para-aortic lymph nodes OR—Odds ratio, CI—confidence interval.

**Table 4:** Multivariate Cox Regression (Stratified by Age for PFS and TFI).

Variables	PFS		TFI		OS	
	H.R (95% CI)	*p* Value	H.R (95% CI)	*p* Value	H.R (95% CI)	*p* Value
Age (≥50 vs. <50)					0.8841 (0.2265–3.4511)	0.8592
BMI	1.1273 (1.0306–1.2331)	0.0089	1.1265 (1.0299–1.2320)	0.0092	1.1032 (0.9948–1.2234)	0.0628
Tumor size (cm)	0.9577 (0.744–1.2333)	0.7379	0.9534 (0.7403–1.2279)	0.7117	0.7697 (0.5383–1.1006)	0.1514
PLN or PALN (pos vs. neg)	2.2967 (0.8143–6.4774)	0.1160	2.2934 (0.8128–6.4714)	0.1168	1.6624 (0.4415–6.2586)	0.4524
PALN (pos vs. neg)	3.5948 (1.6288–7.9339)	0.0015	3.6127 (1.6376–7.9699)	0.0015	3.7172 (1.3036–10.5994)	0.0141
SCC Ag	1.0056 (0.9962–1.0152)	0.2424	1.0058 (0.9963–1.0153)	0.2320	1.0107 (1.0005–1.0211)	0.0398

PFS—Progression free survival, TFI—Treatment Free Interval, OS—Overall Survival, H.R—Hazards ratio, C.I—Confidence interval, PLN—Pelvic lymph nodes, PALN—Para-aortic lymph nodes, BMI—Body mass index, SCC Ag—serum squamous cell carcinoma Antigen, neg-negative, pos—positive.

## Data Availability

Data is unavailable due to privacy restrictions.

## Supplementary Materials

The following supporting information can be downloaded at: https://www.mdpi.com/article/10.3390/cancers17183042/s1, Table S1: Adverse events in the treatment group (N = 128); Table S2: Stratifying analysis as per time period (before and after 2017).
